# A framework for evaluating the impact of the IUCN Red List of threatened species

**DOI:** 10.1111/cobi.13454

**Published:** 2020-01-13

**Authors:** Jessica Betts, Richard P. Young, Craig Hilton‐Taylor, Michael Hoffmann, Jon Paul Rodríguez, Simon N. Stuart, E.J. Milner‐Gulland

**Affiliations:** ^1^ Silwood Park Campus Imperial College London Buckhurst Road Berkshire SL5 7PY U.K.; ^2^ Durrell Wildlife Conservation Trust Les Augres Manor Jersey JE3 5BP Channel Islands; ^3^ IUCN Global Species Programme David Attenborough Building Pembroke Street Cambridge CB2 3QZ U.K.; ^4^ Zoological Society of London Regents Park London NW1 4RY U.K.; ^5^ IUCN Species Survival Commission Instituto Venezolano de Investigaciones Científicas and Provita Caracas Venezuela; ^6^ Synchronicity Earth The Malthouse 17‐20 Sydney Buildings Bath BA2 6BZ U.K.; ^7^ Department of Zoology University of Oxford 11a Mansfield Road Oxford OX1 3SZ U.K.; ^8^ Current address: Fauna & Flora International David Attenborough Building, Pembroke Street Cambridge CB2 3QZ U.K.

**Keywords:** amphibian, conservation funding, counterfactual, impact evaluation, theory of change, anfibios, evaluación del impacto, financiamiento de la conservación, hipótesis de contraste, teoría del cambio, 变化理论, 影响评估, 两栖类, 保护资金, 反事实

## Abstract

The International Union for Conservation of Nature (IUCN) Red List of Threatened Species, a species extinction risk assessment tool, has been guiding conservation efforts for over 5 decades. It is widely assumed to have been instrumental in preventing species from moving closer to extinction and driving recoveries. However, the impact of the IUCN Red List in guiding conservation has not been evaluated. We conducted, transcribed, and coded interviews with experts who use the IUCN Red List across a range of sectors to understand how the list is used in conservation. We developed a theory of change to illustrate how and why change is expected to occur along causal pathways contributing to the long‐term goal of the IUCN Red List and an evaluation framework with indicators for measuring the impact of the IUCN Red List in generating scientific knowledge, raising awareness among stakeholders, designating priority conservation sites, allocating funding and resources, influencing development of legislation and policy, and guiding targeted conservation action (key themes). Red‐list assessments were the primary input leading to outputs (scientific knowledge, raised awareness), outcomes (better informed priority setting, access to funding and resource availability, improved legislation and policy), and impact (implemented conservation action leading to positive change) that have resulted in achievement of IUCN Red List goals. To explore feasibility of attributing the difference made by the IUCN Red List across themes, we studied increased scientific knowledge, raised awareness, access to funding and resource allocation, and increased conservation activity. The feasibility exploration showed increased scientific knowledge over time identified through positive trends in publications referring to the IUCN Red List in the literature; raised awareness of the list following high IUCN activity identified by peaks in online search activity; an increased proportion of conservation funding bodies requesting IUCN Red List status in the application process; and, based on interviews with Amphibian Specialist Group members, red‐list assessments were essential in connecting relevant stakeholders and ensuring conservation action. Although we identified the IUCN Red List as a vital tool in global conservation efforts, it was challenging to measure specific impacts because of its ubiquitous nature. We are the first to identify the influence of the IUCN Red List on conservation.

## Abstract 2

*Article impact statement*: The IUCN Red List has direct and indirect impact on conservation practice; the extent of its influence should be better recognized.

## Abstract (es)

Un Marco de Referencia para la Evaluación del Impacto de la Lista Roja de Especies Amenazadas de la UICN

**Resumen**

La Lista Roja de Especies Amenazadas de la Unión Internacional para la Conservación de la Naturaleza (UICN), una herramienta para la valoración del riesgo de extinción de las especies ha guiado los esfuerzos de conservación durante más de cinco décadas. A partir de esto, se ha asumido como generalidad que la lista ha sido determinante para prevenir que las especies se acerquen más a la extinción y para impulsar la recuperación de especies. Sin embargo, todavía no se ha evaluado el impacto que la Lista Roja de la UICN ha tenido en las directrices de la conservación. Realizamos, transcribimos y codificamos entrevistas con expertos que usan la Lista Roja de la UICN en una gama de sectores para así entender cómo se usa la lista para la conservación. Desarrollamos una teoría de cambio para ilustrar cómo y por qué se espera que ocurra un cambio en Atorno a las vías causales que contribuyen al objetivo de largo plazo que tiene la Lista Roja de la UICN. También formulamos un marco de trabajo con los indicadores necesarios para evaluar el impacto que tiene la Lista Roja de la UICN en la generación de conocimiento científico, la creación de conciencia entre los actores sociales, la designación de sitios prioritarios para la conservación, la asignación de fondos y recursos, la influencia sobre el desarrollo de la legislación y de las políticas, y la orientación de acciones de conservación enfocadas (temas clave). Las evaluaciones de la lista roja fueron los aportadores principales que derivaron en resultados (conocimiento científico, creación de conciencia), consecuencias (establecimiento de prioridades mejor informadas, acceso a financiamiento y disponibilidad de recursos, mejorías en la legislación y en la política) e impactos (acciones implementadas de conservación que derivaron en cambios positivos) que han resultado en la obtención de objetivos de la Lista Roja de la UICN. Para explorar la viabilidad de la atribución de la diferencia que genera la Lista Roja de la UICN en todos los temas clave estudiamos el incremento en el conocimiento científico, la conciencia generada, el acceso a financiamientos y la asignación de recursos, y el crecimiento en las acciones de conservación. Esta exploración de la viabilidad mostró un incremento con el tiempo del conocimiento científico, identificado por medio de tendencias positivas en las publicaciones de la literatura referidas a la Lista Roja de la UICN. También mostró una mayor conciencia por la lista tras una alta actividad de la UICN, la cual identificamos por medio de picos en la actividad de búsqueda en línea. Finalmente, la exploración arrojó una proporción crecida de organizaciones de financiamiento para la conservación que solicitaron el estado de la especie en la Lista Roja de la UICN durante el proceso de aplicación y, con base en entrevistas realizadas a miembros del Grupo Especialista en Anfibios, que las valoraciones de la lista roja fueron esenciales para conectar entre sí a los actores relevantes y para asegurar las acciones de conservación. Aunque identificamos que la Lista Roja de la UICN es una herramienta vital para los esfuerzos mundiales de conservación, fue todo un reto medir los impactos específicos debido a su naturaleza ubicua. Somos los primeros en identificar la influencia que tiene la Lista Roja de la UICN sobre la conservación.

## 摘要

《国际自然保护联盟 (IUCN) 濒危物种红色名录》是一项物种灭绝风险评估工具, 在半个世纪以来指导着保护工作。虽然人们普遍认为它在防止物种走向灭绝和促进物种恢复中发挥着重要作用, 但对于其在指导生物多样性保护中的影响却从未有过评估。我们对多个行业部门使用 IUCN 红色名录的专家进行了访谈, 记录内容并数字化分析, 以探究物种名录在保护中是如何被使用的。我们还提出了一个变化理论来阐述通过因果联系影响 IUCN 红色名录长期目标的变化发生背后的机制和理由, 并设计了一个评估框架, 通过指标衡量 IUCN 红色名录在积累科学知识、提升利益相关者保护意识、确定优先保护位点、分配资金和资源、影响法律和政策制定, 以及指导有针对性的保护行动 (关键主题) 中起到的作用。红色名录评估是导致产出 (科学知识、保护意识) 、结果 (更好地确定优先保护地区、获得资金和资源、改进立法和政策) 和影响 (促成积极变化的保护行动实施) 的主要投入, 它可以帮助实现 IUCN 红色名录目标。为了探索 IUCN 红色名录不同主题差异来源的可能性, 我们进一步分析了科学知识积累、保护意识提升、资金获得及资源分配, 以及保护行动的增加。结果表明, 随着时间推移, 已发表文献对 IUCN 红色名录的引用呈增长趋势, 科学知识也在不断积累; IUCN 行动后相关在线搜索量急剧增加, 说明人们对其认识有所提升; 越来越高比例的保护资助机构在资金申请过程中要求使用 IUCN 红色名录濒危等级; 此外, 两栖动物专家小组成员也在采访中指出, 红色名录评估对沟通利益相关者和保障保护行动的实施至关重要。虽然本研究肯定了 IUCN 红色名录是全球保护工作的重要工具, 但由于应用十分普遍, 难以衡量具体的影响。本研究是首个确定 IUCN 红色名录对保护影响的工作。**【翻译: 胡怡思; 审校: 聂永刚】**

## Introduction

The International Union for Conservation of Nature (IUCN) Red List of Threatened Species is the leading authority on global species extinction risk (Rodrigues et al. 2006; IUCN 2019). During its 5 decades, the IUCN Red List has developed from a subjective list of threatened species compiled by a relatively small group of experts to a scientifically robust, rigorously applied assessment of species extinction risk and threat status based on quantitative criteria and categories (Mace et al. 2008). To date, IUCN Red List assessments have been completed to identify extinction risk for over 105,000 species across several whole major taxonomic groups, including birds (BirdLife International 2017), mammals (Schipper et al. 2008), amphibians (Stuart et al. 2004), and reef‐building corals (Carpenter et al. 2008). In an attempt to resolve the underrepresentation of hyperdiverse species groups, Stuart et al. (2010) set an ambitious target to reach 160,000 IUCN Red List assessments by 2020.

The overall goal of the IUCN Red List is to provide information and analyses on the status, trends, and threats to species to inform and catalyze action for biodiversity conservation. This goal has 2 subgoals: identify and document species facing the highest extinction rates and provide a global index of the state of change of biodiversity by using IUCN Red List data to identify and monitor trends in species threat status. To achieve these goals, IUCN aims to establish a baseline from which to monitor the change in status of species, provide a global context for the establishment of conservation priorities at the local level, and monitor the status of a representative selection of species that cover the major ecosystems of the world (IUCN Red List Committee 2017).

As a highly respected source of information, the IUCN Red List influences many aspects of conservation (policy development, awareness raising, priority setting, resource allocation [Collar 1996; Rodrigues et al. 2006; Hoffmann et al. 2008]). Its reputation is built upon the collaboration of multidisciplinary experts, including members of IUCN's Species Survival Commission (SSC), red‐list partner organizations, IUCN members, universities, museums, research institutes, nongovernment organizations (NGOs), governments, and conservation practitioners across the world. The IUCN Red List assessment process requires assessors to follow scientifically rigorous guidelines and assign any species (excluding microorganisms) to 1 of 8 categories of extinction risk according to an objective set of criteria, based on data linked to population trend, size, and structure and geographic range and their trends over time (IUCN 2012).

It is widely assumed that the development and implementation of the IUCN Red List has led to positive conservation results, and the list is frequently referred to as one of the most influential tools in conservation (Rodrigues et al. 2006). However, with rare exceptions (e.g., Jarić et al. 2017), this influence has not been systematically measured. Monitoring and evaluation allows measurement of a project or program's success toward a desired outcome and impact, while increasing accountability, transparency, and cost‐effectiveness (Salafsky & Margoluis 1998). Despite this, sufficiently robust monitoring and evaluation is typically not carried out in conservation, often because it is considered time‐consuming, resource intensive, or of low priority (Ferraro & Pattanayak 2006). Given the all‐encompassing, complex, and long‐running nature of the IUCN Red List, designing an appropriate evaluation framework that allows changes to be reliably quantified and attributed to the existence and operations of the IUCN Red List is challenging.

Typical approaches to evaluation that enable robust attribution of impact require appropriate indicators to be identified, against which improvement can be measured. However, the impact of the IUCN Red List is a result of multiple, complex, and interrelated factors, making typical evaluation approaches difficult to apply. Process tracing is one method that works well in these circumstances (Collier 2011; Woodhouse et al. 2016). This approach uses qualitative information to evaluate an intervention and provide insight into causal mechanisms through comparisons of hypotheses based on a theoretical scenario (i.e., a theory of change, literature review, or past experience) and the current scenario (Salafsky et al. 2002). During the evaluation process, it is critical to consider a counterfactual scenario in which the intervention did not take place (e.g., What might have happened if the IUCN Red List had not been developed?) to ensure the full impact of the intervention can be measured.

We sought to lay the groundwork for measurement and ongoing monitoring of the impact of the IUCN Red List on species conservation by developing a theory of change that simplifies and helps one visualize the interrelated potential changes brought about by the IUCN Red List. We used this to devise an evaluation framework and sets of indicators to assess the impact across the 6 identified themes and scales and gathered preliminary evidence of the IUCN Red List's impact through quantitative and qualitative case studies on a subset of indicators. We did not seek to assess the relative merits of the IUCN Red List against other threatened species categorization systems or processes, for which previous studies (e.g., De Grammont & Cuaron 2006; Harris et al. 2012) provide an existing evidence base.

## Methods

Following an introduction to our study, we asked experts at the 2016 meeting of the IUCN Red List Committee from a range of sectors, including Red List Committee members, conservation NGO members, members of funding bodies, and those engaged in conservation policy to participate in an interview. These interviews aimed to develop a deeper understanding of how the IUCN Red List was used in conservation and whether the list was perceived as having a positive, neutral, or negative impact on global species conservation. We asked interviewees about their relationship to the IUCN Red List (i.e., red‐list committee member, red‐list assessor, or red‐list user), whether they use the IUCN Red List directly or indirectly and what they believe the biggest impact of the list is. We used snowball sampling to identify other interviewees with different stakeholder roles across sectors and regions. Interviews were conducted until new information regarding impact themes stopped emerging. Interviews were conducted in accordance to Imperial College London's Human Research Ethics protocol.

We transcribed and coded responses to identify frequently discussed themes that could be represented as elements of a theory of change. Three scales were identified through the interviews, and the impact themes were broken down depending on which of these scales they acted: species, major taxonomic group or regional; or global. We developed a conceptual theory of change by mapping the ways in which causality could be assumed between elements along the activity and input–outcome–output–impact continuum. The long‐term impact goal of the IUCN Red List aligns with the impact‐oriented Convention on Biological Diversity Aichi Biodiversity Target 12: “the extinction of known threatened species has been prevented and their IUCN Red List conservation status, particularly of those most in decline, has been improved and sustained.” We then developed an evaluation framework consisting of outputs and outcomes, indicators, assumptions, and methods across each element and scale and considered a counterfactual scenario against which a fuller representation of the impact of the IUCN Red List could be obtained (details in Supporting Information).

The 6 broad elements identified through the theory of change and evaluation framework were: increased derived scientific knowledge; raised awareness of conservation issues; better understood conservation priorities and planning; more or better targeted funding and resource allocation; legal and policy development or change; and more or better targeted conservation action. Of the 56 indicators we developed (Supporting Information), we examined 4 that were in the scope of our study to test IUCN Red List impact: global‐scale increases in scientific knowledge, awareness of conservation, funding and resource allocation and increases in species‐scale conservation action.

The literature citing the IUCN Red List increased each year from 1989 to 2005 (Hoffmann et al. 2008). We hypothesized this trend would continue and the volume of scientific publications in which the IUCN Red List was used as a source of information would increase each year. We used Web of Science to identify trends in publications in peer‐reviewed journals of articles containing the search term “red list” or “red data book” in the title, abstract, or keywords from 1 January to 31 December 1989–2017 (date last checked 31 January 2019). We repeated this search in Google Scholar, which searches whole documents of gray literature and peer‐reviewed articles. With the counterfactual (i.e., the IUCN Red List ceased to be relevant to conservation), we expected the number of articles and documents referencing or citing the IUCN Red List to level off over time. We used this as a counterfactual rather than, for example, the general trend in the amount of conservation literature because we were not interested in the relative growth of the field, but in the actual use of red lists in conservation.

The IUCN Red List generates significant public interest, for example, through media attention or through status displays in educational materials of zoos, aquariums, and botanic gardens. We hypothesized that the volume of information shared online increases over time and reaches increasingly wider audiences, which then increases search activity for the IUCN Red List. This increase was expected to be most apparent following particular IUCN Red List events or campaigns (e.g., 2008 Global Mammal Assessment). Using Google Trends, we examined the popularity of the search term “red list” relative to the total search volume over time. If media attention generated through the IUCN Red List was not reaching a wider audience, we expected no change in frequency of searches for “red list” over time or regions.

The IUCN Red List is frequently referred to as a key influence in donor funding. For example, the Global Environment Facility (GEF) includes information from the IUCN Red List in its System for the Transparent Allocation of Resources (STAR) (Möhner & Klein 2007; Vié et al. 2009). The value of the IUCN Red List to donors and funding bodies is in its ability to divide all species into assessed versus unassessed and to divide assessed species into 8 categories in a transparent and value‐neutral way. Donors interested in threatened species conservation can allocate their resources to species most in need of conservation action, in potential need, or requiring further research. Key conservation funding streams were identified in key‐informant interviews and through an online search (funding bodies supporting “biodiversity conservation” or “wildlife conservation” as their main purpose), which continued through snowball sampling until new information stopped emerging. Following a review of grant application guidelines, we categorized species‐focused funding bodies by maximum size of available grants (small, US$<5,000; medium, US$5,000–49,999; large, US$>50,000), and whether funding applications required applicants to list a species’ IUCN Red List status, species threat status determined by an alternative mechanism, or whether this was not requested. The counterfactual is that, in the absence of the list, funding decisions would rely on other risk ranking protocols or some other mechanism.

The IUCN Red List is considered helpful in prioritizing species for conservation attention and for action to prevent them moving closer to extinction. We hypothesized that following the completion, or reassessment, of an IUCN Red List assessment that placed or uplisted a species in a threatened category, the species or group of species would receive increased conservation attention (e.g., habitat protection, captive breeding). To explore this, we selected the Amphibian Specialist Group (an IUCN SSC Specialist Group with completed IUCN Red List assessments across all species) and developed a timeline depicting the chain of events leading to conservation action. We conducted semistructured key‐informant interviews with red list authority coordinators to determine key events that followed the assessments and whether and how this ultimately led to increased knowledge, awareness, resource allocation, and conservation action for amphibians. The counterfactual was no change in conservation attention to amphibians following the Global Amphibian Assessment or a focus of conservation on species or groups not in IUCN Red List threat categories.

## Results

**Figure 1:**
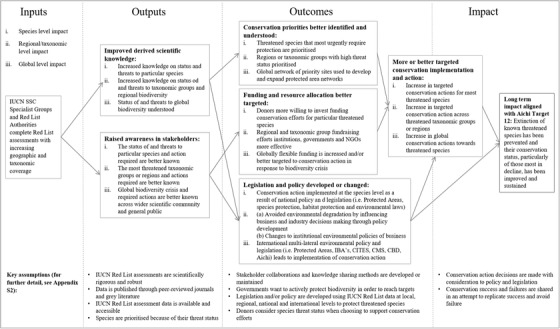
Draft theory of change mapping the interrelated nature of impact elements that contribute to the overarching International Union for Conservation of Nature (IUCN) Red List impact goals (SSC, Species Survival Commission; NGO, nongovernmental organization; IBA, Important Bird and Biodiversity Area; CITES, Convention on International Trade in Endangered Species; CMS, Convention on the Conservation of Migratory Species of Wild Animals; CBD, Convention on Biological Diversity).

No new significant information from experts was retrieved after 28 informal discussions with stakeholders (Supporting Information). Six broad elements of a theory of change were identified: improved scientific knowledge (14 respondents identified scientific knowledge as a key impact of the IUCN Red List); raised awareness of conservation issues (7); better understood conservation priorities and planning (13); more or better targeted funding and resource allocation (7); legal and policy development or change (24); and more or better targeted conservation action (12). The conceptual theory of change, based on the information given in the interviews, defined how the 6 themes were perceived as interrelated and lead to long‐term goals (Fig. 1).

**Figure 2:**
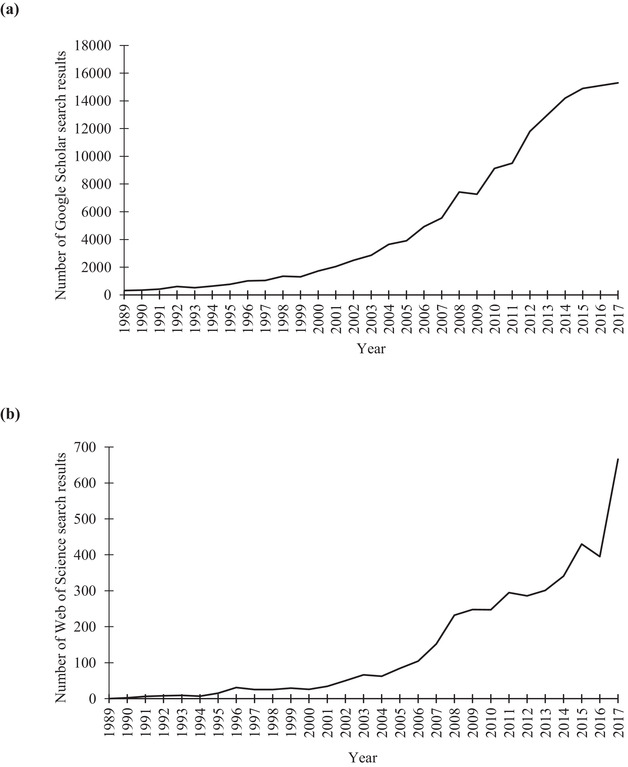
Trend in number of returns when searching for the term “red list” or “red data book” in (a) Google Scholar searches of whole articles of gray literature and (b) Web of Science searches of title, keyword, and abstract of peer‐reviewed literature from 1989 to 2017.

Searches for “red list” or “red data book” identified similar positive trends in peer‐reviewed journals and gray literature from 1989 to 2017, indicating an increase in scientific knowledge generated through the completion of IUCN Red List assessments and national red‐list assessments and the general availability of IUCN Red List data (Fig. 2). Number of mentions in peer‐reviewed articles increased from 0 in 1989 to 666 in 2017 and gray literature pieces increased from 314 in 1989 to 15,300 in 2017.

**Figure 3:**
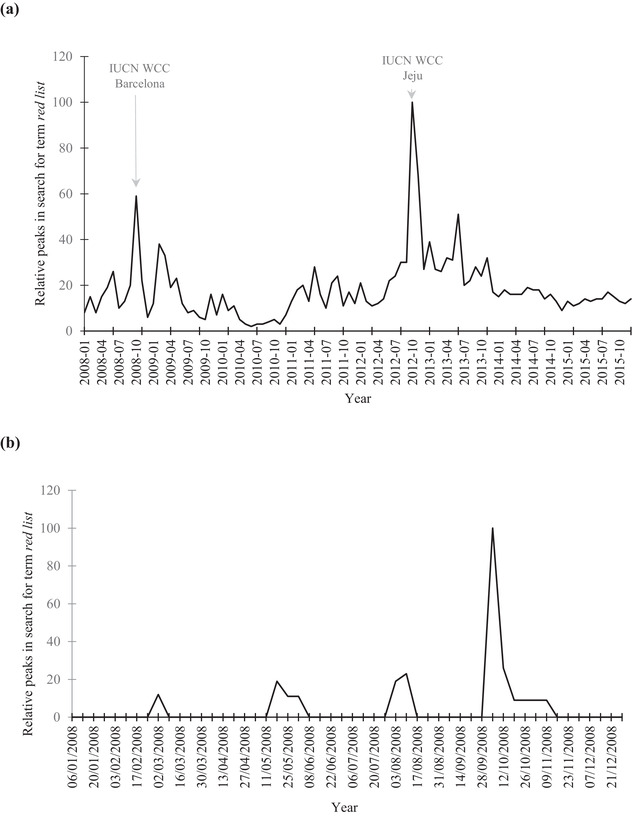
Largest relative peaks in searches for the term “red list” in the News Search filter on Google Trends from (a) January 2008 to December 2015 (October 2008 relative score 59; October 2012 100; November 2012 68; June 2013 51) (IUCN, International Union for Conservation of Nature; WCC, World Conservation Congress; Barcelona, 2008 meeting of WCC; Jeju, 2012 meeting of WCC) and (b) 1 January to 31 December 2008 (weeks commencing 2 March, relative score 12; 18 May, following completion of IUCN Global Coral Assessment, relative score 19; 10 August, following IUCN great apes press release, relative score 23; 5 October, following the WCC meeting in Barcelona and the release of the Global Mammal Assessment, relative score 100).

Google Trend data for “red list” begins in 2008 and continues to the present. The 2 largest peaks in “red list” returns from the News Search filter from 2008 to 2015 were in October 2012 and 2008 following the IUCN World Conservation Congresses (WCC) held in Jeju (relative score 100) and Barcelona (relative score 58), respectively. Peaks in attention following the WCC or red‐list events also occurred over shorter periods. For example, in 2008 there were peaks in search activity that coincided with the release of the Global Mammal Assessment (Schipper et al. 2008) at the WCC, release of the Global Coral Assessment (Carpenter et al. 2008), and a press release about great apes from the IUCN (Fig. 3).

We identified 41 species‐focused funding bodies (Supporting Information). A much higher proportion of species‐focused funding bodies and donors requested applicants state the IUCN Red List status of the focal species (66%) than funding bodies that requested threat status based on an alternative mechanism (29%) or did not request threat status (5%) (Table 1). These results held true regardless of maximum grant size; medium grants had the highest proportion requiring IUCN Red List status be declared (74% vs. 67% for small and 54% for large). Key‐informant interviews revealed the importance of the IUCN Red List in enabling a more strategic and targeted allocation of resources (e.g., “Having the [IUCN] Red List as a scientifically correct and impartial tool is crucial for making funding decisions” [conservation funding manager]). However, interviewees also suggested that without the establishment of the IUCN Red List, alternative mechanisms would have been developed as a means of targeting resources (e.g., [The IUCN Red List] makes it easier to get funding… It would be more difficult to allocate resources without the red list; we would need NGOs and partners to beat the drums of conservation” [collection manager]).

**Table 1:** Size of grants available, number of funding bodies or donors with species‐targeted funding, and percentage of each sized grant requesting IUCN Red List status through application procedures

Grant size (US$)	No. of funding bodies	Funding applications requesting IUCN red list status	Percentage of total funds	Funding applications requesting alternative threat status	Percentage of total funds	Funding applications that do not require species threat status	Percentage of total funds
Small (<5,000)	9	6	66.7	2	22.2	1	11.1
Medium (5,000–49,999)	19	14	73.7	5	26.3	0	0
Large (>50,000)	13	7	53.9	5	38.5	1	7.7
Totals	41	27	65.8	12	29.3	2	4.9

**Figure 4:**
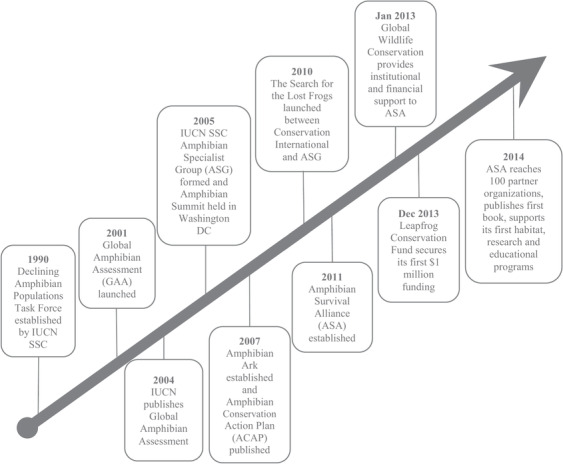
Timeline of events leading to amphibian conservation action following the completion of International Union for Conservation of Nature (IUCN) Red List of Threatened Species assessments contributing to the Global Amphibian Assessment (SSC, Species Survival Commission; ASA, Amphibian Survival Alliance).

Interviewees stated that, following the rigorous application of IUCN Red List categories and criteria, targeting of threatened species for conservation increases to prevent them from moving closer to extinction. We identified several key steps leading to increased conservation following assessment. The conservation community only started engaging in amphibian conservation following the red‐listing process (Stuart et al. 2004), which was completed 15 years after declines were first recorded in 1989. This led to an expansion in the number of organizations focusing on amphibian conservation (e.g., Threatened Amphibian Programme of The Endangered Wildlife Trust established in 2012) and in amphibian conservation organizations (e.g., Amphibian Ark established in 2007). It is expected that there would have been increasing knowledge as scientists continued to identify the rapid and global declines in amphibian populations, but this would not have resulted in the global amphibian conservation work being conducted today had not the red listing of all amphibians been completed and publicized in 2004. The need for communication between red‐list authorities, species experts, academics, funders, governments, and conservation practitioners was repeatedly highlighted as a crucial requirement for successfully securing resources, influencing policy, and ultimately implementing conservation action plans (e.g., “The (IUCN) Red List brings the threat of species to the attention of the right stakeholders, which enables the right people to communicate” [SSC Red List Authority Coordinator]). The completion of the Global Amphibian Assessment triggered a series of events that increased research (Supporting Information) that identified habitat loss and disease as the main threats to amphibians. It also secured funding for amphibian‐focused conservation through the Leapfrog Conservation Fund, which directs funds to support conservation of amphibian species listed as threatened by IUCN. Without the Global Amphibian Assessment, it would be reasonable to assume these funds (among others, such as the Rainforest Trust) might not have been mobilized to such an extent. The Global Amphibian Assessment also increased interdisciplinary collaboration and commitment to amphibian conservation between the Amphibian Survival Alliance and 100 partner organizations and supported research, education, and habitat programs (Fig. 4).

Although discussed in interviews, a specific indicator was not developed and tested for the priority setting and conservation planning or policy and legislation elements. However, red‐list data were perceived by interviewees as essential criteria in priority setting and conservation planning. Foremost among these is the identification of Key Biodiversity Areas (KBA), sites that contribute significantly to the global persistence of biodiversity. Under KBA Criteria and Thresholds, sites that qualify for KBA status “hold a significant proportion of the global population size of a species facing a high extinction risk and so contribute to the global persistence of biodiversity at generic and species level. Species that can trigger criterion A1 encompass those assessed as globally Critically Endangered, Endangered or Vulnerable on the IUCN Red List of Threatened Species” (IUCN 2016). It is also important to consider the role of the IUCN Red List in avoiding or mitigating biodiversity loss through infrastructure development. This is evidenced by the incorporation of the IUCN Red List in the International Finance Corporation (IFC)’s Performance Standard 6: Biodiversity Conservation and Sustainable Management of Living Natural Resources. The IFC is the largest global development institution focused on the private sector in developing countries, and performance standard 6 states that “In areas of critical habitat, the client will not implement any project activities unless it is demonstrated that: The project does not lead to a net reduction in the global and/or national/regional population of any Critically Endangered or Endangered species over a reasonable time period, as listed on the IUCN Red List of Threatened Species.”

The development of legislation and policy was the most frequently discussed impact theme of the IUCN Red List, but the most complex to evaluate. Key‐informant interviewees revealed difficulties associated with using the IUCN Red List in legislative decisions of cross‐boundary species: “We have a particular issue with cetaceans. Some are listed as threatened globally [on the IUCN Red List] on the basis of decline criteria, but if they have not declined in some areas they are not threatened locally. Countries that consider them not declined are actively loath to treat them as threatened” (SSC member). However, the limitations of the IUCN Red List are considered largely irrelevant to policy makers: “A lot of criticisms come from scientists because they think the [IUCN] Red List is not perfect…but to officials the fact that it's imperfect is almost irrelevant, they accept the limitations and use the Red List as a measurable tool….don't let the best be the enemy of the good” (conservation scientist).

## Discussion

Measuring the effectiveness of a widely used conservation tool in general, and the IUCN Red List in particular, is clearly challenging. The variety of ways in which the IUCN Red List influences conservation at a range of scales, in both direct and indirect ways, means it is practically impossible to fully disentangle its role from other influential factors to measure the difference it makes in each impact theme and the interactions between them. Nonetheless, we developed a theory of change of how the IUCN Red List can lead to positive results for conservation and potentially informative indicators that could be used to monitor its influence in the future, foster better appreciation of its role within conservation, and possibly generate more support.

Without a system of robust, quantitative categories and criteria and the input of the IUCN SSC Specialist Groups and Red List Authorities in completing red‐list assessments, the IUCN Red List would not be the objective and scientifically credible measure of extinction risk that it is today. The positive trend in references to “red list” and “red data book” in both scientific and gray literature confirms that the use of red‐list status as a proxy for increasing knowledge about species extinction risk is increasing and people are using the IUCN Red List as a source of information (Stuart et al. 2010). This rapid increase, especially in gray literature, demonstrates the ubiquitous nature of the IUCN Red List, indicating the potential of the list to significantly influence communication and awareness raising of species extinction risk to the scientific community and wider audiences. As demonstrated using the Global Amphibian Assessment, it is possible that data collated through a red‐list assessment can lead to a chain of events that trigger considerable conservation action and even resource mobilization. This collation of scientific knowledge that happens as part of the red‐listing process appears to be instrumental in developing criteria that determine the selection of conservation priority sites and in deciding where to allocate limited conservation resources and where to develop or change legislation. These suggest that enabling the Barometer of Life to reach its goal of 160,000 species by 2020 would be a beneficial investment (Stuart et al. 2010). This benefit could be particularly noticeable in currently underrepresented hyperdiverse species groups (e.g., fungi) for which conservation action is limited.

As the flagship product of IUCN, the Red List is widely recognized in both the scientific community and among the general public, with hundreds of new articles published, reaching thousands of readers through varied media platforms (websites, social media, newspapers, etc.). The results from Google Trends indicated an increasing global awareness of the IUCN Red List that interacts with broader awareness of the IUCN as an organization. Peak online interest in the IUCN Red List occurred following the release of results of annual updates or major global species assessments (e.g., Global Mammal Assessment) and international IUCN meetings (e.g., quadrennial World Conservation Congress). Key informants also revealed the importance of including IUCN Red List materials presented at zoos, aquaria, and botanic gardens in raising public awareness. The power of the IUCN Red List to arouse public imagination is important and must be explored because awareness raising is an important first step in influencing behavior change (Harrison et al. 2000). Using the IUCN Red List to raise awareness of conservation issues locally, regionally, and globally might influence an individual or organization to support biodiversity conservation (e.g., volunteering time to conservation projects or providing financial support to the IUCN Red List). Further research to explore the most effective ways of engaging individuals or organizations in the IUCN Red List is needed to track how this engagement may lead to behavior change.

Key‐informant interviews confirmed the importance of the IUCN Red List as a tool for helping to identify key sites for the persistence of biodiversity, such as KBAs and BirdLife International's global Important Bird and Biodiversity Area (IBA) sites. However, because regional and subregional IBA sites can be determined using other priority species, differences in resource allocation or legal protection may occur. Further research is required to understand the overlap in geographic range, species, resource allocation, and legislation between IUCN‐Red‐List‐selected IBA sites and KBAs more broadly and sites selected using other mechanisms. The IUCN Red List data are also beneficial in conservation planning, for example when industries (i.e., petrochemical and mining) want to offset their negative environmental impact through no net loss and net positive impact initiatives (Bennun et al. 2018). The IUCN Red List categories and criteria are enshrined in environmental safeguard mechanisms (e.g., the IFC's Performance Standard 6 and the World Bank), and it is expected that this role of the IUCN Red List has mitigated further biodiversity loss. It would be useful to explore the extent to which IUCN Red List data are used in global safeguarding mechanisms and the degree to which it has influenced decision making (Burgass et al. 2018).

Our analyses suggested that IUCN Red List status is more frequently requested in applications for species‐focused funding streams than alternative threat classification schemes (e.g., species listing on the U.S. Endangered Species Act) or where threat status is not required. This may be because the IUCN Red List is generally regarded as scientifically rigorous and free of political or other interferences and because, unlike alternative threat determination mechanisms, it reflects the global extinction risk of species. Although the IUCN Red List appeared to streamline resources toward species most at risk of extinction, care must be taken when using it in resource allocation decisions or priority setting decisions (Possingham et al. 2002; Miller et al. 2006; IUCN 2012). The IUCN Red List was not developed as a tool for targeting resource allocation, but as an independent measure of extinction risk. Other variables are also key in priority setting including endemism, taxonomic uniqueness, and existing funding programs and legislation. The development of an IUCN Green Status of Species, which extends the IUCN Red List to include the impact conservation has made, and the potential futures for species with and without ongoing conservation investment should provide further information for informing resource allocation (Akçakaya et al. 2018). However, because the initiative is still in its infancy, it will be some time until the IUCN Green Status of Species achieves the taxonomic breadth needed to inform such efforts. It would be interesting to further analyze the depth of detail required from species focused funding bodies relating to the extinction risk of the species or specific projects to which it intends to allocate resources and to research how resource allocation decisions would be made independent of the IUCN Red List. Currently, no alternative mechanism appears to be as independent or scientifically robust as the IUCN Red List, indicating funding decisions would be much more “difficult and opaque” (fund manager) without it. Similarly, future research would be beneficial to further understand how the gathering of new knowledge about a species immediately following red listing results in up‐ or downlisting.

The impact of the IUCN Red List in policy and legislation development is difficult to evaluate even though the list is intrinsically linked to the development of some multilateral agreements, such as the Convention on International Trade in Endangered Species (CITES). Certainly, the biological criteria used to determine whether a species warrants listing on a CITES appendix draws directly from the IUCN Red List criteria; hence, it could be stated that in the absence of the development of the IUCN Red List, the criteria for the inclusion of a species in the CITES Appendices might look very different. However, elucidating more direct influences is more challenging. Many species currently included in the CITES appendices are not listed on the IUCN Red List or predated the inclusion of the species on the Red List. Further, while the IUCN Red List can certainly inform future amendments to CITES appendices, species listed as threatened that can be linked to international trade may not meet the criteria for inclusion in CITES, particularly when international trade is not a major threat (Challender et al. 2019). The IUCN Red List also forms the basis for a number of biodiversity indicators being used by the CBD, for example to measure progress toward Aichi Target 12 (Butchart et al. 2010), and is likely to be similarly used within the post‐2020 Global Biodiversity Framework (Mace et al. 2018). This is because the IUCN Red List is one of the very few comprehensive, globally available data sets that can be used to track change in biodiversity status; it is difficult to assess what would have been used in its absence. Further research into testing appropriate impact indicators is essential to understand the role of the IUCN Red List in legislative decision making.

A key part of the IUCN Red List's long‐term goal is to “catalyse action for biodiversity conservation.” It is believed IUCN Red List data are essential in guiding conservation action (Rodrigues et al. 2006), and we used the Global Amphibian Assessment to illustratively trace this presumed pathway. There was a chain of events that occurred following IUCN Red List assessment indicating the assessments can lead to conservation action, albeit still greatly insufficient. However, even with the species information generated through an assessment, without successful communication between species experts, academics, policy makers, funders, and practitioners, conservation action plans are unlikely to be developed or implemented.

Among the experts we interviewed across multiple sectors, there was little doubt that the IUCN Red List has made a positive difference to the conservation of biodiversity, and we identified 6 main linked elements that have led to this impact. Through the development of a theory of change and evaluation framework, this research is the first step in systematically identifying the impact of the IUCN Red List. To further assess whether the IUCN Red List is achieving its long‐term goal, and the return on financial investment that it represents, more indicators need to be incorporated into the framework, and these need to be systematically tested.

## Supporting information

A list of informal discussion and interview participants (Appendix S1), evaluation framework (Appendix S2), list of species‐focused funding bodies (Appendix S3), citation rate of Stuart et al. (2004) (Appendix S4), additional manuscript information (Appendix S5), and list of questions used in the interviews and discussions (Appendix S6) are available online. The authors are solely responsible for the content and functionality of these materials. Queries (other than absence of the material) should be directed to the corresponding author.
