# Supplementary material for: A framework for evaluating the impact of the IUCN Red List of threatened species
Source: Conserv Biol. 2020 Jan 13;34(3):632–43. doi: 10.1111/cobi.13454 (PMC7318271; doi:10.1111/cobi.13454)
Supplement: Supplementary file 1 — A list of informal discussion and interview participants (Appendix S1), evaluation framework (Appendix S2), list of species‐focused funding bodies (Appendix S3), citation rate of Stuart et al. (2004) (Appendix S4), additional manuscript information (Appendix S5), and list of questions used in the interviews and discussions (Appendix S6) are available online. The authors are solely responsible for the content and functionality of these materials. Queries (other than absence of the material) should be directed to the corresponding author. [file COBI-34-632-s001.docx]

Supporting Information

Appendix S1. Informal discussion and interview participants.

Appendix S2. IUCN Red List evaluation framework.

Appendix S3. Species focused conservation funding sources.

Appendix S4. Stuart et al. 2004; citation rate.

Appendix S5. Additional manuscript information.

Appendix S6. Questions used in interviews and discussions.

Appendix S1. Informal discussion and key informant interview participants

| Angulo, Ariadne | IUCN SSC Amphibian Specialist Group |
| --- | --- |
| Bennett, Liz | Wildlife Conservation Society |
| Bohm, Monika | Zoological Society of London |
| Butchart, Stuart | BirdLife International |
| Clausnitzer, Viola | Senckenberg Museum of Natural History |
| Cooke, Justin | Centre for Ecosystem Management Studies, Germany |
| Donald, Paul | BirdLife International |
| Gardner, Laura | Zoological Society of London |
| Heard, Nicolas | Mohamed bin Zayed Species Conservation Fund |
| Hilton-Taylor, Craig | IUCN |
| Hoffmann, Mike | IUCN |
| Hoffmann, Rachel | IUCN |
| Joppa, Lucas | Microsoft |
| Keith, David | University of New South Wales |
| Long, Barney | Global Wildlife Conservation |
| Luedtke, Jennifer | Global Wildlife Conservation |
| Mace, Georgina | University College London |
| Mallon, David | Manchester Metropolitan University |
| McGowan, Philip | Newcastle University |
| Milner-Gulland, E.J. | University of Oxford |
| Norris, Ken | Zoological Society of London |
| Paul Rodriguez, Jon | IUCN |
| Raghavan, Rajeev | Kerala University of Fisheries and Ocean Studies |
| Rodrigues, Ana | Centre d’Ecologie Fonctionelle et Evolutive |
| Secoy, Katherine | Zoological Society of London |
| Smart, Jane | IUCN |
| Stanley Price, Mark | University of Oxford |
| Stephenson, PJ | WWF |
| Stuart, Simon | IUCN |
| Terry, Andrew | Durrell Wildlife Conservation Trust |
| Thomas, Philip | Royal Botanic Garden Edinburgh |
| Vincent, Amanda | University of British Columbia |
| Young, Richard | Durrell Wildlife Conservation Trust |
|  |  |

Appendix S2. IUCN Red List evaluation framework

|  | **Scale 1: individual species or individual species populations (i.e. geographically from local to national to global scale).** | **Scale 2: Whole taxonomic groups and/or regional communities of species (i.e. at national level, ecoregion level).** | **Scale 3: IUCN Red List impact on threatened species as a whole at a global level.** |
| --- | --- | --- | --- |
| **Improved**  **Derived**  **Scientific**  **Knowledge** | *Outcomes/Outputs:*   - IUCN SSC Specialist groups continue to input time and resources to complete Red List Assessments for previously unassessed species. - Specialist Groups conduct re-assessments of species at regular intervals to ensure most current data is available. - New data is generated to fill knowledge gaps in particular species or particular species populations. | *Outcomes/Outputs:*   - Gaps in knowledge about species extinction risk in taxonomic groups or regions are filled to better understand distribution and traits of Data Deficient species. - Specialist Groups have plans in place to conduct further assessments and reassessments of species in their group. - The use of data generated through Red List Assessments for conservation purposes is made available through paper publications in peer-reviewed. - Red List Assessment data is published in scientific magazines/blogs etc. and is accessible to institutions. - The wider scientific community is aware of the completion of Red List   Assessments through paper publications in peer-reviewed journals. | *Outcomes/Outputs:*   - International NGO’s and governments continuously conduct research and generate scientific knowledge through conservation projects and this data then feeds into the IUCN Red List. - Supporting data generated through Red List Assessments leads to the development of new conservation tools to understand global trends in biodiversity (i.e. Red List Index and Sampled RLI, Living Planet Index). - International NGO’s and governments are aware of and have access to conservation tools. - The number of Red List Assessments being completed each year increases in recent years in an attempt to reach   160,000 target by 2020.* |
|  | *Indicator:*   - Number of additional Red List assessments completed - Number of Red List re-assessments completed | *Indicator:*   - Number of Data Deficient species reassessed - Action plans are developed - Number and frequency or articles published through social media, | *Indicator:*   - Number of papers published in peer-reviewed journals, scientific reports and environmental reports |

|  | - Number of Red List assessments completed to fill gaps in knowledge           *Assumptions*:  The information produced by completing a Red List Assessment is made available through supporting documentation. The Red List Assessment supporting documentation is freely available and easily accessible online.  Red List Assessments increase and enhance scientific knowledge rather than just collating it. | newspapers, websites, magazines, blogs   - Number of papers published in peer-reviewed journals       *Assumptions*:  When a Red List Assessment is completed, Specialist Groups publish the data in peer-reviewed journals.  Conservation projects which use Red List Assessment data in their conservation efforts publish papers in peer-reviewed journals and scientific magazines/blogs. | - Number of paper published regarding conservation tools developed through the Red List - Rate at which Red List assessments are being completed     *Assumptions:*  Interdisciplinary experts communicate and collaborate to develop conservation tools and then use these tools in conservation efforts. Papers using conservation tools are published in peer-reviewed journals.  Conservation tools are cited in increasing numbers of papers in peer-reviewed journals. Experts communicate with NGO’s and governments.  Global species experts collaborate and engage with Specialist Groups to assess and reassess more species in order to reach the 160,000 target by 2020. |
| --- | --- | --- | --- |
|  | *Method:*  Use the Red List website to identify the number of Red List Assessments and reassessments being completed each year. Use the Red List website to ensure supporting material for Red List Assessments are available and accessible online. Identify trends in number of Red List assessments being completed each year. | *Method:*  Use Web of Science to identify number of papers published in peer-reviewed journals as a result of Red List Assessments being completed and as a result of Red List Assessments being used to implement conservation action.  Identify changes in use of Red List data over time by filtering search results on Web of Science.  Use Google Scholar to identify the number of articles, theses, books and abstracts that are available because of Red List Assessments. | *Method:*  Use global databases, peer-reviewed journals and government reports to identify any change in the number of occasions Red List tools are used.  Use Web of Science and Google Scholar to look at trends over time in the publication and citation of Red List tools in journals, articles, books, theses and abstracts.  ***Case Study**: Use Web of Science and Google Scholar to calculate the number of papers, articles, abstracts, theses and books published each year from 1989 (58 in total) to 2015 (86,323 in total) with only 135 new publications in 2001 compared to 1577 new publications in 2015 alone. |

|  | *Counterfactual/attribution:*  Action plans for species assessments to be completed independent of the IUCN Red List are in place. | *Counterfactual:*  The number of Red List Assessment papers being published in peer-reviewed journals in no different to the number of papers being published relating to conservation on non-Red Listed species. | *Counterfactual:*  The development and uptake of Red List tools has not influenced conservation decision making. |
| --- | --- | --- | --- |
| **Raised**  **Awareness** | *Outcomes/Outputs:*   - Specialist Groups and NGO’s run campaigns and develop educational material about a threatened species or population. - Members of the public have access to material through news articles, blogs, posters, leaflets etc. - The general public and wider scientific community are aware of the Red List threat status of a particular species or population. | *Outcomes/Outputs:*   - NGO’s and governments use IUCN Red   List data to produce articles, blog posts and educational material to raise awareness of threatened endemic species and in-country wildlife.   - Zoos, aquaria and botanical gardens hold events to attract members of the public to increase awareness of nationally or regionally threatened species. - Wider scientific community and general public are aware of the work being completed by IUCN Specialist Groups to assess and re-assess species for the IUCN Red List. | *Outcomes/Outputs:*   - International IUCN campaigns raise awareness of the Red List and its activity globally (i.e. Red List 50). - International governments and NGO’s are aware of and use the data generated through Red List Assessments. - Red List extinction risk information is provided at zoos, aquariums and botanical gardens globally. - Education programmes are used to teach children, young people and public about conservation efforts of threatened species globally. - The Red List is actively discussed online with articles being shared through websites and social media platforms.* |
|  | *Indicator:*   - Number of articles relating to particular species following awareness raising campaign. - Articles published reach wider scientific community and general public audiences | *Indicator:*   - Number of articles relating to whole groups (e.g. amphibians, vultures) - Number of participants engaging in public awareness events | *Indicator:*   - Number of articles published relating to the IUCN Red List increases - Articles published reach wider scientific community and general public audiences |

|  | *Assumptions:*  Information about particular species extinction (using the Red List status as a proxy) is made available on platforms accessible to the public. | *Assumptions:*  At the regional and national levels, members of the public demonstrate an interest in understanding more about threatened wildlife.  Resources are available to zoos, aquariums and botanical gardens to hold public events. | - Number of individuals that participate and engage with education programmes     *Assumptions:*  When knowledge is generated through the Red List, experts will make the information accessible through articles and blogs published on NGO or government websites. Information provided by the IUCN about the Red List is spread online.  Zoos, aquariums and botanical gardens demonstrate extinction risk of species within their displays and enclosures.  As awareness of the Red List increases, more information is shared in the general public and scientific community. |
| --- | --- | --- | --- |
|  | *Method:*  Use Google Trends to look at how Google searches for different species change over time following their Red List Assessment or Reassessment.  Use social media platforms to identify the reach and distribution of particular media. | *Method:*  Use the IUCN Red List and Specialist Group web pages to look at changes in the number of webpage hits following the completion of Red List Assessments by Specialist Groups. Use Google Trends to identify changes in frequency of searches following particular  Red List events (i.e. completion of Global Amphibian Assessment, Red List 50 campaign).  Use Google Trends to look at changes in search activity by region or taxonomic group. Use Altmetric to look at the total online activity surrounding a species, region or threat category search term across multiple websites and social media platforms. | *Method:*  Contact zoos, aquariums and botanical gardens to understand how Red List information is used and displayed to inform and engage visitors.  Interview members of zoo, aquarium or botanical garden education programmes to understand how the development, availability and uptake of education programmes have changed over time (i.e. ZSL Education Department).  Research publicly accessible NGO and governmental articles or documents which relate to Red List data.  Use Altmetric to look at total online activity surrounding the term “Red List” across multiple websites or social media platforms.  ***Case Study**: Google Trend data from 2008 shows four peaks in the number of times the term “Red List” was searched, which corresponds with IUCN Red List activity. |

|  | *Counterfactual:*  Members of the public and wider scientific community learn about species threats through NGO’s or governments, regardless of IUCN Red List. | *Counterfactual:*  Members of the public and the wider scientific community use information from other mechanisms (i.e. US Endangered Species Act, EU Birds Directive) to better understand conservation issues. | *Counterfactual:*  Awareness is related to other traits of species including threats or biology independent of the IUCN Red List (e.g. Rainforest species, widely distributed species, hunted species). |
| --- | --- | --- | --- |
| **Priority Setting** | *Outcomes/Outputs:*   - IUCN SSC Specialist Groups identify most threatened species within their Specialist Group for prioritisation. - Species most threatened with extinction are prioritised for increased conservation attention (resource allocation, legal protection etc.) after the completion of a Red List Assessment compared to species which are not categorised as threatened or have not been assessed. - Individual populations of threatened species are recognised and particularly vulnerable populations are prioritised. | *Outcomes/Outputs:*   - IUCN SSC Specialist Groups are formed when groups of species are widely threatened either regionally or taxonomically (i.e. Amphibian Specialist Group development following the Global Amphibian Assessment). - Experts and institutions develop criteria to determine regions of high extinction risk using data produced through Red List Assessments. - Experts and institutions implement action plans to reduce threats and minimise extinction risk or further decline of prioritised species/groups. | *Outcomes/Outputs:*   - Governments and international NGO’s invest time and resources in identifying species of particular importance and global areas of high extinction risk for prioritisation using data generated through the IUCN Red List (i.e. EDGE species or KBA/IBA sites). - Communication is maintained between   Specialist Groups, NGO’s and governments to ensure high priority areas are identified and protected.   - Industries explore opportunities to reduce the negative impacts on biodiversity and promote more sustainable production - Initiatives of petrochemical, mining aggregate and financial industry such as Net Positive Impact (NPI) and No Net Loss benefit from access to information on the distribution of species and their conservation status. |

|  | *Indicator*:   - Action plans developed place for priority species - Attention focussed on particularly threatened species               *Assumptions:*  Species and/or populations are prioritised because of their threat status. | *Indicator:*   - Number of Specialist Groups formed to tackle species groups threats - Criteria and guideline documents for priority site selection - Documents outlining action plans for species or groups of species           *Assumptions*:  Global network of experts identify when groups of species are threatened through research and completion of Red List Assessments.  Experts collaborate to evaluate risk and develop action plans for the protection of priority areas and species. | *Indicator*:   - Number of prioritisation mechanisms that are underpinned by IUCN Red List data - Partnerships with industry - Number of industry organisations which use initiatives such as NPI or No Net Loss - Rate of uptake of NPI and No Net Loss initiatives by industries     *Assumptions*:  Experts regularly communicate most recent research and data with each other and with international NGO’s and governments. Governments want to actively protect priority species and areas as it will assist in reaching global biodiversity targets (i.e. Aichi Target 12 for the Convention on Biological Diversity). |
| --- | --- | --- | --- |
|  | *Method:*  Use the Sampled Red List to identify groups of species which have been partially assessed and then identify assessed species which are in a threatened category.  Use ‘before and after control intervention’ design to draw comparisons in conservation effort between non-assessed species, non-threatened species and threatened species. Use Key Informant Interviews with Red List assessors to understand the differences in conservation attention received by threatened vs. non-threatened and non-assessed species. | *Method:*  Use Key Informant Interviews with members of Specialist Groups to understand how Red List Assessments change the way species are prioritised depending on the category to which they are assigned.  Use Key Informant Interviews with local governments to understand if and how they use priority sites in legislation. | *Method:*  Use Key Informant Interviews with international NGO’s, governments or Red List Authorities to understand the extent to which the Red List is used in determining priority sites.*  Use Key Informant Interviews with environmental departments to understand how development and extraction planning procedures use Red List information.  ***Case Study**: Key Informant Interviews with BirdLife (IUCN Red List Authority for birds) to understand how the Red List is integrated in their criteria to identify globally Important Bird and Biodiversity Areas. |

|  | *Counterfactual:*  Species which are not listed on the IUCN Red List are protected through other priority mechanisms (i.e. US Endangered Species Act, EU Birds Directive). | *Counterfactual:*  Local governments do have prioritisation sites but these are based on a different set of criteria to the Red List.  Species action plans are developed and implemented using different priority criteria. | *Counterfactual:*  Biodiversity hotspots are still identified and protected from development and extraction through national criteria or legislation (i.e.  US Endangered Species Act). |
| --- | --- | --- | --- |
| **Funding and**  **Resource**  **Allocation** | *Outcomes/Outputs:*   - IUCN Red List status is a key factor driving individual donation decisions. - Particular species or populations receive funding attention because of their threatened Red List status (i.e.   Vulnerable, Endangered and Critically Endangered).   - Individual donors and funding bodies allocate resources to the conservation efforts for specific threatened species (i.e. African Elephant Conservation Fund). | *Outcomes/Outputs:*   - Individual donors and funding bodies commit to funding ecoregions or taxonomic groups with high proportion of threatened species (i.e. amphibian group/Cape Floristic Region). - Governments use the IUCN Red List to prioritise resource allocation to threatened endemic species or national populations of threatened species. - Donations and resources made to taxonomic groups or ecoregions are used directly in implementing conservation action rather than administrative costs etc. - Specialist Groups with an active member body (re/assessment plans, conservation action plans, actively seeking funding) receive more resources to protect threatened species. | *Outcomes/Outputs:*   - Funding is made available to threatened species conservation efforts globally through large scale international funding streams (World Bank, GEF).* - Funding bodies use Red List data to inform decisions on where to allocate resources. - International communication is maintained between funding bodies to ensure resources are allocated to subpopulations or migratory species across whole ranges. - Members of the public donate directly to the IUCN Red List through the Red List website and through other means. - Long term funding streams are established for global threatened species conservation. - Global resources are better targeted because of the IUCN Red List. |

|  | *Indicator:*   - Amount donated to conservation efforts of specific threatened species - Amount donated to threatened species in comparison to species which are not - Proportion of funding available to specific species           *Assumptions:*  Donors choose to support conservation efforts for particular species based on their threat status.  The more threatened a species is, the more funding it will receive in comparison to a non-threatened or non-Red Listed species. | *Indicator:*   - Amount available to conservation efforts of threatened taxonomic groups or regions - Government funding available only to threatened species or populations - Proportion of donations made to conservation efforts vs. other costs - Amount of funding received by specific Specialist Groups     *Assumptions:*  More active Specialist Groups engage with more donors and are given more funding. Donors prefer to support taxonomic groups or ecoregions where they are most likely to see a return on investment.  Governments want to protect national populations or species. | *Indicator:*   - Amount of funding to conservation made available by international funding bodies - Funding and grant applications require indication of Red List status for target species - Partnerships between international funding bodies - Proportion of globally flexible funding committed to long term projects     *Assumptions:*  Funding bodies want to prioritise their resources to species most at risk of global extinction and will therefore consider IUCN Red List status in decision making. Bigger conservation benefits will be seen when funding Critically Endangered or Data Deficient species. |
| --- | --- | --- | --- |
|  | *Method*:  Research how frequently specific species conservation funds require a declaration of species threat status.  Identify the amount of funding made available through the donations web page on the Red List website.  Conduct literature review to understand how donors support species based on threat status. | *Method:*  Use Key Informant Interviews with governments, national conservation projects and Specialist Groups to understand where their funding comes from and how much is tied to the average IUCN Red List status of their species groups. | *Method:*  Use Key Informant Interviews with donors to understand how they use threat status information in decision making.  Research how frequently funding sources are only available to species in specific IUCN  Red List threat categories.*  ***Case Study**: Use grant making body application forms to identify how frequently global funding and resources are only available to conservation efforts of threatened species because of IUCN Red List status. |

|  | *Counterfactual:*  Species which are not on the Red List still receive targeted funding.  Species which are on the Red List receive funding because of other forms of prioritisation (i.e. EDGE species). | *Counterfactual:*  NGO’s working with species which are not Red Listed still receive funding for conservation attention. | *Counterfactual:*  Resource allocation decisions would be made using expert opinion or alternative prioritisation mechanisms (e.g. US Endangered Species Act, EU Birds Directive). |
| --- | --- | --- | --- |
| **Legal and**  **Policy**  **Change or**  **Development** | *Outcomes/Outputs:*   - Policy and legislation decisions are made at the local, regional, national and international level using data generated through the IUCN Red List to protect specific threatened species. - Threatened species are legally protected from direct threats (i.e. hunting) and indirect threats (i.e. habitat degradation and loss). - Threatened species with legal protection see an improvement in conservation status, or a reduction in threat pressure. | *Outcomes/Outputs:*   - Policy and legislation are developed to protect threatened species in taxonomic groups or regions using the IUCN Red List. - Specialist Group members provide expert knowledge to governments to allow most informed policy and legislation decisions to be made. - NGO’s use national policy information to influence conservation action decisions of endemic species and national populations. - Governments generate National Red Lists based on the categories and criteria of the IUCN Red List. | *Outcomes/Outputs:*   - Specialist Groups and NGO’s place lobbying pressure on governments to protect globally threatened species or habitats. - International collaboration allows data generated through Red List Assessments to be intrinsically included in international policy (i.e. CITES, CBD, CMS). - Tools developed through the Red List (i.e. Red List Index, Sampled RLI) are used in policy decision making.  International networks of NGO’s use international policy to influence conservation action decisions for globally threatened species. |
|  | *Indicator:*   - Proportion of species specific legislation that used Red List data in development - Number of laws or policies introduced to protect threatened species - Number of species prevented from moving closer to extinction or improving as a result of specific policy or legislation | *Indicator:*   - Partnerships between Specialist Groups and governments - Number of laws and policy specifically in place to protect threatened endemic species or populations - Criteria guideline documents for National Red Lists | *Indicator*:   - Legislation and policy developed because of lobbying pressure - Number of international policies and conventions which incorporate Red List data into their indicators - Number of policies and conventions that use tools generated through the Red List as indicators |

|  | *Assumptions*:  Local, regional, national and international governments want to generate legislation to protect threatened species as it will assist in reaching global conservation targets. Governments will benefit from strengthened relationships with conservation NGO’s and projects through policy development. Species which are listed as most threatened will have the strongest legal protection and therefore see best conservation outcomes. | *Assumptions:*  Regional areas and taxonomic groups with high levels of extinction risk will receive highest legal protection.  Communication is maintained between policy makers, species experts and NGO’s. Conservation action decisions are made with consideration to policy and legislation. | - Number of international NGO conservation action plans which incorporate international policy     *Assumptions:*  Policy decision makers value the IUCN Red List as an independent, scientifically robust database of extinction risk. Data generated through Red List Assessments are used in policy decision making. |
| --- | --- | --- | --- |
|  | *Method:*  Select a random sample of species, of which some have been Red List Assessed and some have not, and identify using literature searches and Key Informant Interviews how much legal protection each species receives. Draw comparisons between the amount of legal protection for species in threatened categories and those which are not.  Use Key Informant Interviews with species experts to identify any change in behaviour following introduction of legislation (i.e.  reduced hunting). | *Method:*  Use Key Informant Interviews with experts in Specialist Groups and NGO’s to understand the amount of legal protection in a given region or taxonomic group.  Use Key Informant Interviews with NGO’s to understand how decisions are made using existing policy. | *Method*:  Using web searches, identify how frequently international biodiversity conventions and targets are based upon data generated through Red List Assessments, or use tools derived from the Red List.  Use Key Informant Interviews with local and national government policy makers to understand how Red List data is used in informing legislative decisions. |

|  | *Counterfactual:*  Species are legally protected through legislation which was developed before or in parallel to the IUCN Red List. | *Counterfactual:*  NGO’s use threat data independent of IUCN Red List data to influence decisions.  Where independent National Red Lists exist, governments do not use the IUCN Red List to influence policy and legislation decisions. | *Counterfactual:*  Species are legally protected through legislation which was developed before or in parallel to the IUCN Red List. |
| --- | --- | --- | --- |
| **Conservation**  **Action** | *Outcomes/Outputs:*   - Red List Assessments accurately identify direct and indirect threats of specific species.* - Threat mitigation efforts for specific species are implemented through conservation NGO’s, projects and other conservationists. - Threatened species benefit from in- and ex-situ conservation efforts. | *Outcomes/Outputs:*   - Specialist Group members and Red List Assessors provide expert knowledge to conservation practitioners. - Taxonomic and regional experts collaborate with species experts to develop in-country conservation action plans for endemic species and threatened species populations. - In-country conservation practitioners   (IUCN partners, NGO’s, academics, governments) use information and action plans to implement in- and ex-situ conservation (i.e. habitat protection or restoration, captive breeding programmes, reintroduction projects). | *Outcomes/Outputs:*   - Multi-disciplinary experts collaborate and commit to developing projects to protect globally threatened biodiversity. - Knowledge is shared between conservation practitioners, governments,   NGO’s and academics to allow successful implementation of projects across species international ranges, or to overcome similar threat risks.   - An increased proportion of globally threatened species are protected through international conservation efforts. |
|  | *Indicator:*   - Threats clearly identified in Red List assessment information - Threat mitigation incorporated in species conservation action plans - Change in species abundance or threat status following conservation efforts. | *Indicator:*   - Partnerships between Specialist Group members, Red List assessors and   conservation practitioners   - Number of endemic species and threatened population action plans - Documents outlining in- and ex-situ conservation efforts. | *Indicator:*   - Number of projects established as a result of multi-disciplinary collaboration - Partnerships between conservation practitioners, governments, NGO’s and academics - Number and proportion of threatened species receiving conservation attention. |

|  | *Assumptions:*  Red List Assessments are thorough and rigorous.  NGO’s and other conservationists use the Red List as a knowledge database to learn about threat status of particular species to allow implementation of conservation efforts. | *Assumptions:*  Communication is maintained between all conservation implementation stakeholders. Conservation action implementers use the knowledge provided by species and regional experts to develop and target conservation action plans. | *Assumptions:*  When a conservation project experiences success or failure, it is shared so that similar successes can be achieved or failures avoided.  Communication is maintained internationally between conservation practitioners and species experts. |
| --- | --- | --- | --- |
|  | *Method:*  Key Informant Interviews with Red List Assessors and conservation practitioners to identify how much a Red List Assessment influences conservation effort.  ***Case Study:** Key Informant Interviews with  Amphibian Specialist Group and Conifer Specialist Group Red List Authority coordinators to identify changes in conservation action for a species following the completion of a Red List Assessment, or the down listing of a species into a more threatened category. | *Method:*  Key Informant Interviews with Specialist Group members and NGO’s (i.e. Antelope Specialist Group working in collaboration with Marwell Zoo). | *Method:*  Conduct Key Informant Interviews with individuals along the whole spectrum of conservation action from Red List Assessor through to those who implement conservation projects (i.e. Red List Assessors in Specialist Groups, academics, NGO’s, conservation practitioners and governments). |
|  | *Counterfactual:*  Species experts still identify threats to species in the absence of the Red List. There is no difference in the amount of conservation attention received by species that have been Red List assessed and those which have not. | *Counterfactual:*  There is no difference in the amount of resources received by a threatened species when compared to a non-threatened or non-Red Listed species. | *Counterfactual:*  International collaborations allow for expert knowledge to be shared without the presence of the IUCN to facilitate. |

Appendix S3. Conservation funding sources with grant size (S – small, M – medium, L – large).

| 1 | African Bird Club Conservation Awards | S |
| --- | --- | --- |
| 2 | African Bird Club Expedition Awards | M |
| 3 | Amphibian Ark Conservation Extentsion | S |
| 4 | Amphibian Ark Conservation Grant | M |
| 5 | Asian Waterbirds Conservation Fund | M |
| 6 | International Iguana Foundation | M |
| 7 | NatGeo Recovery of Species on the Brink of Extinction | L |
| 8 | National Geographic Big Cats Initiative | L |
| 9 | Neotropical Bird Club - Juan Mazar Barnett award | L |
| 10 | Neotropical Bird Club - medium grant | S |
| 11 | Neotropical Bird Club - small grant | S |
| 12 | Ornithological Society of the Middle East | S |
| 13 | Pacific Seabird Group | S |
| 14 | Primate Conservation Inc | M |
| 15 | Save Our Species Rapid Action Grant | M |
| 16 | Save Our Species Threatened Species Grant | L |
| 17 | Save the Rhino International | L |
| 18 | Sirenian International Protection of Manatees and Dugongs | S |
| 19 | USFWS African Elephant Conservation Fund | L |
| 20 | USFWS Asian Elephant Conservation Fund | L |
| 21 | USFWS Conservaiton of Neotropical Birds | L |
| 22 | USFWS Great Ape Conservation Fund | L |
| 23 | USFWS Marine Turtle Conservation Fund | M |
| 24 | USFWS Rhinoceros and Tiger Conservation Fund | L |
| 25 | ZSL EDGE of existence programme | M |
| 26 | Chicago Zoological Society | M |
| 27 | Columbus Zoo Conservation Grants Program | M |
| 28 | Disney Worldwide Conservation Fund - Annual Conservation Grant | L |
| 29 | Disney Worldwide Conservation Fund - Rapid Response Grant | M |
| 30 | Fondation Segre | L |
| 31 | Fresno Chaffee Zoo - Grants for Wildlife Conservation | S |
| 32 | Future for Nature Foundation | L |
| 33 | Mohamed Bin Zayed Species Conservation Fund | M |
| 34 | Phoenix Zoo | S |
| 35 | Prince Bernhard Nature Fund | M |
| 36 | Riverbanks Zoo and Garden | M |
| 37 | Rufford Foundaiton Grants for Nature Conservation | M |
| 38 | Save our Seas Foundation - Keystone grants | L |
| 39 | Save our Seas Foundation - small grants | M |
| 40 | USFWS Program for Mexico | M |
| 41 | Weeden Foundation Quick Response Biodiversity fund | M |

Appendix S4. Stuart et al. 2004; citation rate**.**

Trend in number of citations of “Status and trends of amphibian declines and extinctions worldwide” in the years following the release of the Global Amphibian Assessment in 2004 (Stuart et al. 2004) from 2005 to 2015.

Appendix S5. Additional manuscript information.

Methods: Theory of Change development (Manuscript; line 144)

We developed a conceptual theory of change by determining whether each element was an input/activity (the initial activity or resources implemented, in this case the resources to complete IUCN Red List assessments), an output (results achieved immediately after application of an activity/input), an outcome (a change which has occurred as a result of an output) or an impact (the broader or longer term change as a result of one or several outputs). Using this categorisation, we began to map the ways in which causality could be assumed along this continuum (e.g. the theory of change suggests that there will be increased knowledge following the completion of a Red List assessment), and indicators were developed as a way of measuring and testing these assumptions. We then developed an evaluation framework consisting of outputs/outcomes, indicators, assumptions and methods, across each element and scale, whilst considering a counterfactual scenario against which a fuller representation of the impact of the IUCN Red List could be obtained.

Results: Conservation action at species scale (Manuscript; line 253)

Interviewees stated that, following the rigorous application of IUCN Red List categories and criteria, threatened species receive more conservation action to prevent them from moving closer to extinction. Using the Global Amphibian Assessment and interviews with members of the IUCN SSC Amphibian Specialist Group, several key steps leading to increased conservation action were identified following Red List assessment. Following key informant interviews, it is evident that in most parts of the world, and especially in the tropical areas where amphibian declines and extinctions have been most severe, the conservation community only started to engage in amphibian conservation following the Red Listing process. The scientific community first started talking seriously about amphibian declines in 1989 at the World Congress in Herpetology, after recording declines in Australia, Costa Rica, and Brazil. The Global Amphibian Assessment, which was completed 15 years later in 2004, highlighted the extent and distribution of these declines, culminating in the publication of Stuart et al. 2004. Much of the information generated through the initial Red Listing process had not been published in peer-reviewed journals at that time, but was unpublished information provided by scientists. As a result, the Global Amphibian Assessment, and in particular the red-listing that resulted from it, revealed the true status of the worldwide amphibian crisis for the first time. Following the 2004 publication, there was an expansion in the number of conservation organisations focusing on amphibian conservation (e.g. Threatened Amphibian Programme of The Endangered Wildlife Trust established in 2012), and an increase in amphibian conservation organisations being established (e.g. Amphibian Ark and Save the frogs Ghana, established in 2007 and 2011 respectively). It is expected that there would have been increasing knowledge as scientists continued to identify the rapid and global declines in amphibian populations, but this would not have resulted in the global amphibian conservation work being conducted today had not the Red Listing of all amphibians been completed and publicized in 2004.

Appendix S6. Questions used in interviews and discussions.

Questions were asked in informal discussion and Key Informant Interviews. They were semi-structured and open ended, giving the participant the opportunity to elaborate on points raised.

What is your relationship to the IUCN Red List (for example, member of IUCN Species Specialist Group, conservation practitioner protecting an IUCN Red List threatened species)?

How long have you had this relationships with the IUCN Red List? Has this changed over time?

Do you perceive the IUCN Red List as having a positive, neutral or negative impact on biodiversity conservation?

Why do you believe the IUCN Red List to have a positive/neutral/negative impact on biodiversity conservation?

In your opinion, what has been the biggest impact of the IUCN Red List on biodiversity conservation?
